# An Evaluation of Phosphate Buffer Saline as an Alternative Liquid-Based Medium for HPV DNA Detection

**DOI:** 10.31557/APJCP.2021.22.11.3441

**Published:** 2021-11

**Authors:** Wiyada Dankai, Surapan Khunamornpong, Sumalee Siriaunkgul, Aungsumalee Soongkhaw, Phanlapa Aithin, Suree Lekawanvijit

**Affiliations:** 1 *Gynecologic Cancer Research Center, Faculty of Medicine, Chiang Mai University, Chiang Mai, Thailand. *; 2 *Department of Pathology, Faculty of Medicine, Chiang Mai University, Chiang Mai, Thailand. *

**Keywords:** Human papillomavirus, liquid-based medium, phosphate buffer saline, HPV DNA detection

## Abstract

**Objective:**

HPV detection has been proposed as part of the co-testing which improves the sensitivity of cervical screening. However, the commercially liquid-based medium adds cost in low-resource areas. This study aimed to evaluate the performance of ice-cold phosphate buffer saline (PBS) for HPV detection.

**Methods:**

HPV DNA from SiHa cells (with 1-2 copies of HPV16 per cell) preserved in ice-cold PBS or PreserveCyt solution at different time points (24, 36, 48, 72, 120 and 168 h) was tested in triplicate using Cobas 4800. The threshold cycle (Ct) values of both solutions were compared. An estimated false negative rate of PBS was also assessed by using the difference in Ct values between both solutions (∆Ct) and Ct values of HPV16-positive PreserveCyt clinical samples (Ctsample) at corresponding time points. Samples with a (Ctsample+∆Ct) value > 40.5 (the cutoff of HPV16 DNA by Cobas 4800) were considered as false negativity.

**Results:**

The Ct values of HPV16 DNA of SiHa cells collected in PBS were higher than PreserveCyt ranging from 0.43 to 2.36 cycles depending on incubation times. There was no significant difference at 24, 72, 120, and 168 h. However, the Ct values were statistically significantly higher for PBS than PreserveCyt at 36 h (31.00 vs 29.26), and 48 h (31.06 vs 28.70). A retrospective analysis in 47 clinical PreserveCyt collected samples that were positive for HPV16 DNA found that 1 case (2%) would become negative if collected in ice-cold PBS.

**Conclusions:**

The PBS might be an alternative collecting medium for HPV detection in the low-resource areas. Further evaluations are warranted.

## Introduction

Cervical cancer is the fourth most common cancer among women worldwide (Sung et al., 2021). In Thailand, cervical cancer is the second most common cancer in women in Thailand (Imsamran et al., 2015; Wilailak and Lertchaipattanakul, 2016). In 2012, 8,184 new cases of cervical cancer and 4,513 deaths in Thai women were estimated by the ministry of public health and ministry of education (Imsamran et al., 2015). According to data from the National Cancer Registry, the age-standardized incidence rate (ASR) for cervical cancer in Thailand was 11.7-14.4 per 100,000 (Imsamran et al., 2015; Kengsakul et al., 2021). Cervical cancer is highly related to human papillomavirus (HPV), especially high-risk HPV (hrHPV) group (Li et al., 2011). Molecular testing has been developed for detecting the HPV viral nucleic acid in clinical samples. The HPV DNA detection with a high sensitivity has improved the performance of cervical cancer screening (Pileggi et al., 2014; Agorastos et al., 2015). Therefore, the HPV DNA detection has been recommended to use with liquid-based cytology as a co-test in the primary cervical cancer screening program (Lorincz et al., 2013).

The Cobas 4800 HPV DNA test was approved by the United States Food and Drug Administration in 2011 for detecting hrHPV DNA. This test is based on real-time polymerase chain reaction (PCR) technology and nucleic acid hybridization that detects 14 hrHPV types: HPV type 16, 18 and a pool of 12 other hrHPV types (HPV 31, 33, 35, 39, 45, 51, 52, 56, 58, 59, 66 and 68) (Cui et al., 2014). Cervical cells were recommended to be collected in ThinPrep PreserveCyt solution, Cobas PCR cell collection media and SurePath preservative fluid. 

PreserveCyt, a widely used preservative for liquid-based cytology in cervical cancer screening (Bolick and Hellman, 1998; Liverani et al., 2006), is a methanol based buffer which can preserve cell morphology as well as DNA for molecular study (Dimulescu et al., 1998; Zhao et al., 2011). 

However, this preservative solution adds a substantial cost for low-resource areas that need hrHPV nucleic acid testing.

Phosphate buffer saline (PBS) is an isotonic solution commonly used in laboratory especially for cell culture application. PBS is nontoxic to living cells and can retain cell viability during transportation (Martin et al., 2006). Due to its economical cost and simple preparation, we hypothesized that PBS may be an alternative collecting medium for HPV DNA detection under temperature and time control. 

Therefore, this study aimed to evaluate whether ice-cold PBS could be used as a collecting medium for HPV DNA detection in SiHa cells at different collected time points, compared with PreserveCyt solution. In case that the performance of ice-cold PBS is inferior to PreserveCyt, an estimated false negative rate of PBS was also assessed by using data from clinical samples that were collected in PreserveCyt and were positive for HPV16 DNA by Cobas 4800 HPV DNA test.

## Materials and Methods

*Cell culture*

The SiHa human cervical cancer cell line containing 1-2 copies of HPV DNA type 16 per cell was cultured in DMEM (Gibco, Life Technologies) supplemented with 10% FBS (Biochrom GmbH, Germany) in T-25 flask (Nunc, Shanghai). Cells were incubated at 37°C in 5% CO_2_ incubator until reaching 90% confluence. 

*SiHa cell preparation for HPV DNA detection*

After harvesting, 2x10^5^ SiHa cells were placed in 2 ml of ice-cold PBS or PreserveCyt solution (Hologic, Inc.) for various incubation times (24, 36, 48, 72, 120 and 168 h). For each time point, both PBS and PreserveCyt samples were prepared in triplicate. Then, HPV DNA detection was performed simultaneously using Cobas 4800 HPV DNA test according to manufacturer recommendations. 

Briefly, samples were processed for DNA extraction using the Cobas X480 instrument then DNA was purified by magnetic beads system. The purified DNA was transferred into the Cobas Z480 for PCR amplification with subsequent nucleic acid hybridization. The human β-globin gene was used as an internal control for the quality of DNA extraction. Test was validated with positive and negative control in each run. Results were interpreted as negative or positive for HPV16, HPV18 or other hrHPVs. Test with no β-globin gene amplification and negative HPV DNA was interpreted as invalid.

*Retrospective analysis for estimating the performance of PBS in clinical samples*

The retrospective study population was part of 5,331 women enrolled in the population-based cervical cancer screening program in Northern Thailand during January 2014 to December 2015. All samples in this program were collected in PreserveCyt and tested for hrHPV DNA by Cobas 4800 HPV DNA test. There were 79 women who were positive for HPV16 DNA. Among these, 47 had time from collection to test at 1, 2, 3, 5 and 7 days. An estimated Ct value of HPV16 DNA if using ice-cold PBS was calculated in each case as follow;

Estimated Ct value = Ctsample + ∆Ct (corresponding time point)

where,

Ctsample = Ct values of HPV16-positive PreserveCyt clinical samples

∆Ct = difference in Ct values between PBS and PreserveCyt solution from the SiHa experiment at corresponding time point

*Ethical considerations*

This study was approved by the institutional ethic committee of the Faculty of medicine, Chiang Mai University in study code: FAC-MED-2561-05275.

*Statistical analysis*

Absolute Ct values from each triplicate experiment were collected. Unpaired t-test was used for comparisons between solutions at each time points using GraphPad Prism software version 7. A P value of < 0.05 was considered statistically significant. 

## Results

*Ct values of SiHa cells at different incubation times*

**Figure 1:**
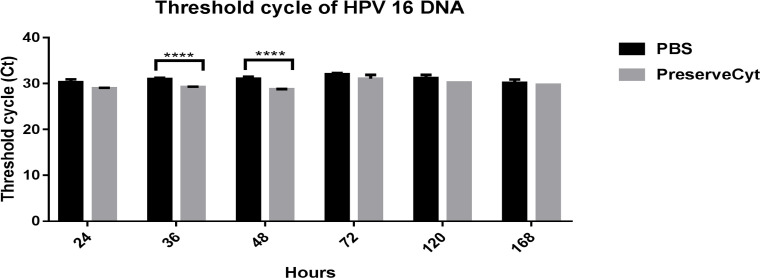
Ct values of HPV16 DNA from SiHa Cell Line at Different Incubation Times (**** p < 0.0005, unpaired t-test)

**Figure 2:**
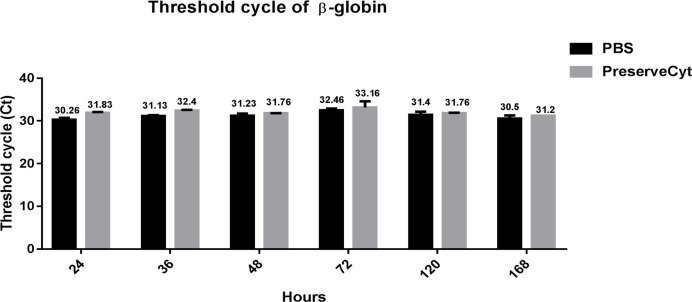
The Ct of β-globin of SiHa Cell Line at Various Time Points between Ice-Cold PBS and PreserveCyt

The Ct values of HPV16 DNA ranged from 30.13 to 32 for ice-cold PBS and 28.7 to 31.06 for PreserveCyt (Table 1). They were 0.43-2.36 cycles (∆Ct) higher in ice-cold PBS than PreserveCyt at all time points. At 36 h and 48 h, the Ct values of HPV16 DNA were significantly higher in ice-cold PBS than PreserveCyt (Figure 1). However, there was no difference at 24 h and during 72 h to 168 h. The Ct values of β-globin gene, an internal control gene, were comparable between PBS and PreserveCyt collected samples at all time points (Figure 2).

*Estimated false negative rate of ice-cold PBS in clinical samples*

**Figure 3:**
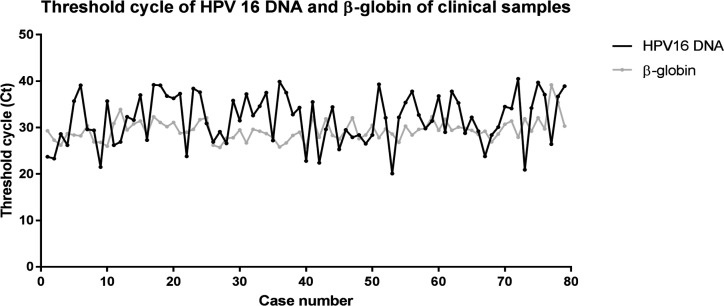
The Ct of HPV16 DNA at Various Time Points of PreserveCyt Collected Clinical Samples

To estimate a false negative rate of ice-cold PBS in clinical samples, 79 women with HPV16-positive PreserveCyt collected samples were retrieved from a total of 5,331 tested samples during January 2014 to December 2015. The Ct values of these 79 samples ranged from 20.1 to 40.5 for HPV16 DNA and 25.7 to 39.2 for β-globin gene (Figure 3). 

Forty-seven of 79 samples had collection-to-test time points corresponding to the SiHa experiment which were 1, 2, 3, 5 and 7 days. Estimated Ct values in these samples if using ice-cold PBS were calculated by summing a ∆Ct at the corresponding time point and Ctsample (Table 2). 

Since the cutoff value of HPV16 by Cobas 4800 HPV DNA test is set at 40.5 (Stoler et al., 2011; Lindemann et al., 2012), any samples with an estimated Ct value higher than 40.5 were considered as a false negative sample. There was only 1 out of 47 samples (2%) expected to be negative for HPV16 in case of using PBS (Table 2). 

**Table 1:** The Ct Values of HPV16 DNA and ∆Ct at Different Incubation Times

Incubation time (h)	Ct values of PBS		Ct values of PreserveCyt		∆Ct*
	Ct	Average	Ct	Average	
24	29.6		28.8		
	30.9	30.26	28.9	28.93	1.33
	30.3		29.1		
36	31.2		29.3		
	30.7	31	29.3	29.26	1.74
	31.1		29.2		
48	30.6		28.7		
	31.3	31.06	28.6	28.7	2.36
	31.3		28.8		
72	32.1		30.5		
	31.7	32	32	31.06	0.94
	32.2		30.7		
120	31.9		30.2		
	31.1	31.16	30.2	30.2	0.96
	30.5		30.2		
168	29.8		29.7		
	29.6	30.13	29.7	29.7	0.43
	31		29.7		

**Table 2:** Estimated Ct values of HPV16 DNA in Case of Using Ice-Cold PBS as a Collection Medium

Collection to test time (Day)	Ct values of HPV16 DNA (Ct_sample_)	∆Ct from SiHa experiment	Estimated Ct values (Ct_sample_ + ∆Ct)
1 (n=5)	20.1	1.33	21.43
	23.8		25.13
	28.8		30.13
	29.8		31.13
	37.1		38.43
2 (n=10)	26.4	2.36	28.76
	29		31.36
	31.5		33.86
	31.6		33.96
	32.6		34.96
	32.7		35.06
	36.7		39.06
	36.7		39.06
	37.6		39.96
	38.9		41.26
3 (n=14)	20.9	0.94	21.84
	23.8		24.74
	25.3		26.24
	26.2		27.14
	29.6		30.54
	29.6		30.54
	30.9		31.84
	31.4		32.34
	35.4		36.34
	35.7		36.64
	35.8		36.65
	37		37.94
	37.8		38.74
	38.4		39.34
5 (n=13)	26.2	0.96	27.16
	26.5		27.46
	26.6		27.56
	27.2		28.16
	27.3		28.26
	28.4		29.36
	29.2		30.16
	32.1		33.06
	32.3		33.26
	34.1		35.06
	34.2		35.16
	34.5		35.46
	35.5		36.46
7 (n=5)	26.9	0.43	27.33
	27.9		28.33
	28.4		28.83
	28.6		29.03
	37.8		38.23

## Discussion

The HPV DNA detection has been used in combination with cervical cytology for primary cervical cancer screening (Lorincz et al., 2013). Liquid-based cytology samples are recommended for most approved HPV DNA detection kits. However, adding cost of commercial collecting media for liquid-based cytology can be substantially problematic in low-resource areas. 

Low-cost liquid medium for HPV DNA detection has been evaluated by Castle et al., (2007). They suggested that the mouthwash was as alternative low-cost liquid medium for storage of cervical specimens for HPV DNA detection (Castle et al., 2007). However, the mouthwashes are available in various and different in the composition depending on the company. 

Since PBS is commonly used in laboratory, cheap, easy to prepare and non-toxic to cells, the present study therefore hypothesized that ice-cold PBS might be used as an alternative cell collecting medium for HPV DNA detection by Cobas 4800 HPV DNA test. 

The present study demonstrated that HPV DNA detection of SiHa cells collected in ice-cold PBS was comparable to the reference liquid-based medium PreserveCyt. For the SiHa experiment, there was no significant difference in Ct value of the internal control β-globin gene between ice-cold PBS and PreserveCyt at every time point. This confirms that ice-cold PBS can preserve the SiHa cells similar to PreserveCyt up to 7 days. The performance of ice-cold PBS appeared to be inferior to PreserveCyt with regard to HPV DNA detection at 36 h and 48 h however there was no significant difference during 72-168 h. 

Given that an increase in Ct value of HPV16 DNA if using ice-cold PBS instead of PreserveCyt ranged from 0.43-2.36 cycles, any sample with HPV DNA copy number of 20.43-2.36 or 1.35-5.13 times of the limit of detection may become negative if using PBS as a collection media. However, the present study also demonstrated the estimated false negative rate of only 2% from a retrospective analysis of clinical samples in the past 2 years. 

Banura et al., (2008) evaluated the performance of PBS in the detection of 25 HPV types compared with paper smear using SPF10 HPV LiPA version 1 (Banura et al., 2008). The prevalence of any type HPV of the cervical exfoliated cells collected in PBS showed much higher than paper smear (82.9% vs. 32.4%; kappa statistic = 0.18) (Banura et al., 2008). 

This study demonstrated that ice-cold PBS could be used to preserve DNA in tissue culture cells for HPV detection. The finding suggests that the PBS is has a potential to be used as a collection media for cervical cancer screening samples, particularly in low-resource settings. As this study was based only on culture cell samples, the comparison of clinical performance in HPV DNA testing between PBS and standard preservative solution was not available. Further study in clinical cervical samples is needed in the comparative evaluation of the performance of PBS including sensitivity, specificity, false positive, and false negative values.

In summary, our study provided preliminary evidence that HPV DNA detection can be performed by using ice-cold PBS. The ice-cold PBS would be beneficial for HPV DNA detection in low-resource areas still using conventional Pap smear because its cost per test was approximately 8 times lower than PreserveCyt (1.41 USD vs 11.41 USD; the present study). Limitation in the use of PBS should be addressed as PBS needs to be preserved in cold temperature and the samples needs to be processed within the time limit (one week after collection). Thus, clinical application of PBS would require a well-organized arrangement. However, PBS is cost-saving compared to the standard collection media for HPV testing, and this can be beneficial to cervical cancer screening in low-income areas.

## Author Contribution Statement

WD: conceptualized and designed the study, conducted data collection, analyzed and interpreted the data, wrote the first draft of the manuscript, and approved the final version to be published; SK: critically reviewed the manuscript; SS: critically reviewed the manuscript; AS: conducted data collection; PA: conducted data collection; SL: conceptualized the study, analyzed and interpreted the data, and critically reviewed the manuscript; All authors reviewed the results and approved the final version of the manuscript.

## Funding Statement

This work was supported by the National Research University Project which was authorized by the Higher Education Commission of Thailand and Faculty of Medicine, Chiang Mai University.
